# Dr. William Hopkins Prudden

**Published:** 1912-09

**Authors:** 


Dr. William Hopkins Prudden, Buffalo, 1905, a resident of N. Y. City, died at the Roosevelt Hospital, July 24, aged 30, after an operation for acute appendicitis.

